# Experimental and Numerical Investigations on the Damage Induced in the Shearing Process for QP980 Steel

**DOI:** 10.3390/ma15093254

**Published:** 2022-04-30

**Authors:** Shuo Han, Ying Chang, Cunyu Wang, Yun Han, Han Dong

**Affiliations:** 1 School of Automotive Engineering, Dalian University of Technology, Dalian 116024, China; hanshuo@mail.dlut.edu.cn; 2 State Key Laboratory of Advanced Special Steel and Shanghai Key Laboratory of Advanced Ferrometallurgy, Shanghai University, Shanghai 200444, China; donghan@shu.edu.cn; 3 Central Iron & Steel Research Institute (CISRI), Beijing 100081, China; wang_cunyu@126.com; 4 Shougang Research Institute of Technology, Beijing 100043, China; hanyun@shougang.com.cn

**Keywords:** shearing process, microvoid nucleation, sheared-edge formability

## Abstract

Ultra-high-strength quenching and partitioning (Q&P) steels have achieved remarkable lightweight effect in automotive manufacture due to the excellent mechanical performances. However, the problem of sheared-edge cracking greatly limits their application. In this work, the damage generated in the shearing process of QP980 steel is experimentally investigated via microstructure characterization and micro-/macromechanical property evaluation. Moreover, the shearing deformation is simulated with six widely used damage models. The experimental results reveal that microvoids, microcracks, and work-hardening behavior are the main damage factors affecting the formability of sheared edges. Microvoids mainly formed at phase interfaces have a small size (≤5 μm), while microvoids generated from inclusions with a small number have a large size (>5 μm). As deformation continuously grows, microvoids distributed around the sheared surface are split into microcracks, which act as crack initiators in the subsequent forming step. Additionally, the highest microhardness in the fracture zone further enhances the susceptibility of edge cracking. Furthermore, the optimum damage model for QP980 steel was determined by developing user-defined subroutine VUSDFLD in Abaqus, which can be used in the prediction of fracture behavior of QP980 steel to reduce the risk of edge cracking.

## 1. Introduction

**Figure 1:**
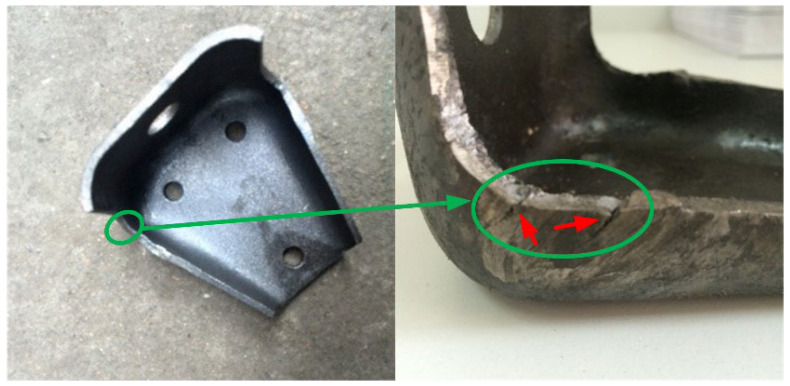
Phenomenon of sheared-edge cracking in the supporting frame part.

Advanced high-strength steels (AHSSs) with improved formability and mechanical performance are developed to meet the demands for greater crash performance while simultaneously reducing mass and cost. The quenching and partitioning (QP) steels are widely considered as one of the most promising candidates for the third-generation AHSSs, which have an excellent balance of high strength and adequate ductility attributed to their multiphase microstructure consisting of a ferrite matrix with martensite and retained austenite phase [1]. At present, the effects of alloy composition, rolling and processing temperatures, and cooling profile have been sufficiently analyzed to widen the range of potential mechanical properties of QP steels. For example, the composition of C-Mn-Si-Al-type QP steel with a strength of 1593 MPa and total elongation of 13% is successfully processed by controlling rolling temperature and strain [2]. The counter-balancing effects of Si on C partitioning have been clarified in a 10 Mn quenching and partitioning steel [3]. Compared with slow gas jet (SJ) cooling, intercritical (IC) annealing can obtain more balanced mechanical properties [4]. A novel QP steel (0.225C–0.85Si–2.02Mn–0.91Al, in wt.%) with the product of strength and elongation (PSE) above 24 GPa% is achieved by high-temperature short-time overaging treatment [5]. The relationship of retained austenite and cooling rate is investigated to obtain a suitable heat treatment [6] Although outstanding mechanical performances of QP steels have been achieved by microstructure control and validated by tensile tests in the laboratory, the issue of sheared-edge cracking of these materials always occurs in industrial production [7,8] and greatly restricts their applications. The typical example of sheared-edge cracking is shown in Figure 1; the cracks resulting from the shearing process are indicated by red arrows in Figure 1.

Plenty of experimental works have been performed on the aspect of optimizing shearing process parameters and developing new shearing methods to reduce the susceptibility of sheared-edge cracking. For example, obvious improvements of sheared-edge formability for the specific AHSSs are realized by experimental trials to determine the optimum shearing process parameters, including shearing clearance [9], shearing angle [10], sheet holder force [11], shearing speed [12], and die failure [13,14]. In addition, new shearing methods, such as smoothing the sheared edge [15], notch-shear cutting [16], electrohydraulic shearing process [17], and two-stage shear cutting [18], have been invented by adding additional procedures. The adequate formability of sheared edges is manifested via these new shearing methods, which can be commonly applied to different steels. However, the experimental trials on the optimum process parameters and design of new shearing methods have the disadvantages of a low efficiency and high cost, which limit the application in the high-efficiency and cost-controlling automotive industry. Therefore, it is necessary to investigate the damage induced in the shearing process in QP steel to obtain the basic understanding of the cause of sheared-edge cracking and adopt reasonable methods to suppress the failure of edge cracking without increasing cost at the same time.

Some researchers investigate the influence of microstructure on the formability of sheared edges. For example, the high fraction and low stability of retained austenite induced by quenching at high temperature reduce the formability of the sheared edge of a quenched and partitioned 0.22C–3.79Mn–1.48Si–0.98Cr (in wt.%) steel, the reason being that the large difference in hardness between the high-C strain-induced martensite and the low-C martensite matrix leads to the formation of cracks at the boundary area [19]. The influence of properties of microstructure constituents of DP and CP steels is also studied, especially the strength of the matrix and the contrast in hardness between softer and harder phases, and the results show that decreasing the hardness between the constituent phases is beneficial for obtaining high sheared-edge formability [20]. Additionally, the effect of the grain size of twinning-induced plasticity (TWIP) steel is also investigated [21,22], and the results show that grain refinement is the best way to develop a high strength and high sheared-edge formability at the same time. From the aspect of microstructure, the key points to improve the sheared-edge formability are to improve the microstructure homogeneity and reduce the grain size.

To decrease the time and cost spent on the experiments for obtaining the optimum shearing process parameters, various ductile fracture criteria are developed to simulate the shearing process. For example, the coupled ductile fracture criteria considering damage accumulation induced by the evolution of microvoids, including the modified Gurson Tvergaard Needleman (GTN) model [23], and Lou–Huh model [24], have been successfully applied in the simulation of the shearing process. Additionally, uncoupled ductile fracture criteria, including the Cockcroft and Latham model [25,26,27], Modified Mohr–Coulomb (MMC) model [28,29,30], and Oyane model [31], have also been employed to characterize the fracture behavior in the shearing process. Compared with the coupled criteria, the uncoupled criteria are preferred for industrial applications due to their simplicity. Some other damage evaluation models are developed to simulate the failure behavior. For example, a unified and generic numerical modeling approach is provided to describe the damage evolution behavior under collapse loads, which is validated with experiment estimates [32]. Numerical simulations using progressive fracture analysis (PFA) and the cohesive zone model (CZM) are realized to assess the loss of load capacity under axial compression load [33]. However, the simulation for the shearing process of QP steel has not been reported yet and the proper ductile fracture criterion has not been determined.

In summary, the problem of the sheared-edge cracking of AHSSs has been studied through experiments and simulations. However, the damage induced in the shearing process and accurate shearing simulation for the QP steel remain unexplored, which are essential to control the phenomenon of sheared-edge cracking. Therefore, the present work determines the mechanical damage generated in the shearing process of QP steel and the damage degree under three shearing clearances is quantitatively analyzed for the first time. Additionally, appropriate uncoupled ductile fracture criterion is determined to simulate the shearing process of QP980 steel, which can be used in the prediction of sheared-edge forming process to avoid edge failure.

The damage induced in the shearing process, namely microvoids, microcracks, and work-hardening behavior, is investigated through microstructural characterization and micro-/macromechanical property evaluation. Additionally, considering that the uncouple ductile fracture criteria are preferable for industry, six different uncoupled ductile fracture criteria are adopted in our study to simulate the shearing process. The simulation results are compared with the corresponding experimental results to determine the suitable ductile fracture criterion. Finally, tensile tests are performed on the samples with sheared edges fabricated under different shearing clearances to evaluate the formability of the sheared edge. As a result, our research contributes to improving the mechanical performance of the sheared edge by identifying and controlling the damage induced in the shearing process. Additionally, the time and cost spent on the experimental trials for the optimum shearing process parameters is reduced through accurate numerical simulations. Therefore, the obtained knowledge can provide guidance for the application of QP980 steel in the automotive body constructures with a complex shape.

## 2. Material and Methods

### 2.1. Material

**Figure 2:**
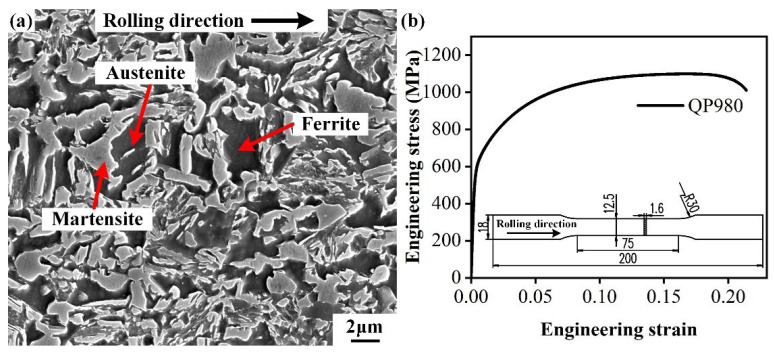
(**a**) Microstructure of QP980 steel. (**b**) Engineering stress–strain curves of QP980 steel.

The investigated material is a commercially produced cold-rolled QP980 steel with a thickness of 1.6 mm. The QP980 steel consists of ferrite, martensite with an irregular shape, and retained austenite with small size, which is determined by the shape and image contrast [34,35,36,37], as depicted in Figure 2a. Among the constituent phases, martensite contributes to the enhancement of strength, ferrite is beneficial to the improvement of ductility, and retained austenite provides a transformation-induced plasticity (TRIP) effect to enhance the strength and ductility simultaneously during the deformation. Therefore, a balanced combination of an adequate total elongation of 21.4% and excellent tensile strength of 1098.7 MPa is exhibited in the tensile test, as shown in Figure 2b and Table 1.

### 2.2. Methods

The shearing process of QP980 steel was studied through a combination of experiments and simulations. Firstly, the original steel sheet is sheared by the self-developed experimental platform. Secondly, the damage induced in the shearing process is investigated using microstructure characterization and microhardness tests. Finally, the formability of the sheared edge of QP980 steel is evaluated by the tensile test. Additionally, the shearing process is simulated in the commercial software Abaqus via developing a user-defined subroutine VUSDFLD. The Abaqus is developed by Dassault Systèmes company in France. The version used in this paper is 2017 and the work of simulation is supported by Supercomputing Center of Dalian University of Technology with Abaqus license.

#### 2.2.1. Shearing Experiment

**Figure 3:**
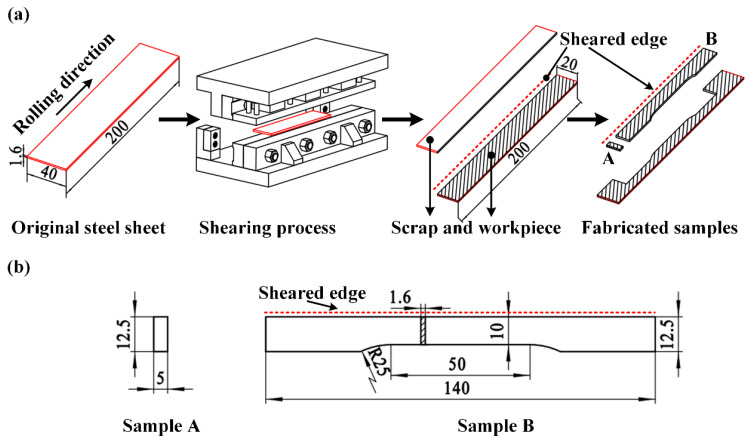
(**a**) Experimental procedure. (**b**) Sample dimensions (all units are in mm).

A self-developed experimental platform was employed in this study, the information about the equipment has been introduced in detail in our previous study [38]. The shearing speed of 30 mm/s was adopted under the shearing clearances of 5%t, 10%t, 15%t (t means thickness), and the specific shearing clearances were 0.08 mm, 0.16 mm, and 0.24 mm, respectively. The detailed experimental procedure and sample dimensions are illustrated in Figure 3. The QP980 steel of 200 mm × 40 mm × 1.6 mm was sheared into two parts with the same size along the rolling direction, and then the sample of workpiece indicated in Figure 3a was fabricated into sample A and sample B by electrical discharge machining (EDM). Sample A was used for the microstructure characterization and microhardness tests, while sample B was used for the tensile test.

#### 2.2.2. Microstructure Characterization

Sample A, shown in Figure 3b, was metallographically prepared by mechanical grinding, polishing, and chemical etching for microstructure analysis using scanning electron microscopy (SEM, FEI Quanta 650 type, Hillsboro, OR, USA). The sample preparation procedure is summarized as follows: First, sample A was hot mounted by the Simplimet-4000 mounting press (Buehler, Lake Bluff, IL, USA). Next, the examining surface was ground with 150#, 320#, 600#, and 1000# silicon carbide grinding in turn. Then, the ground surface was polished by the SG-POL-820 polisher (Shang Guang, Shanghai, China). Finally, the surface was etched with 4% nital for approximately 15 s in order to reveal the microstructure.

**Figure 4:**
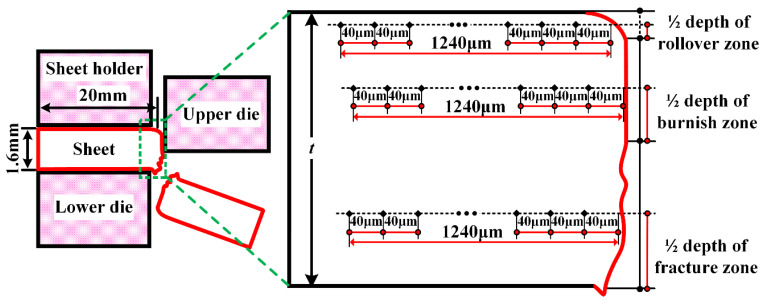
Location of microhardness testing points.

The microhardness test was employed to characterize the work-hardening behavior in the sheared edge according to the international standard ISO 6570-1 [39]. The microhardness in the rollover zone, burnish zone, and fracture zone was tested along the horizontal direction from the sheared surface to matrix under a load of 50 g at a dwell time of 15 s, as shown in Figure 4.

#### 2.2.3. Mechanical Property Tests

**Figure 5:**
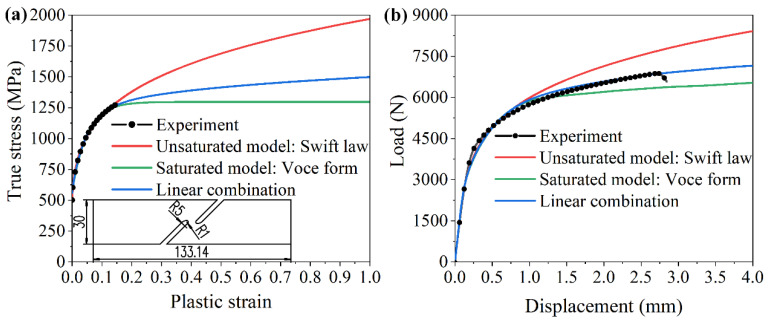
(**a**) True stress–true plastic strain curves. (**b**) Load–displacement curves.

Three kinds of tensile tests performed on an electromechanical testing machine (WDW-300E type, Shijing, Jinan, China) were applied in our study to identify the material parameters and evaluate the mechanical performances of QP980 steel. The uniaxial tensile test controlled at the tensile speed of 2 mm/min was manipulated according to the international standard ISO 6892-1 [40], and the dimensions of the standard dog bone-shaped sample is shown in Figure 2b. The tensile property of sample B with the sheared edge shown in Figure 3b was examined according to the previous method [38]. The shear test determining the stress–strain relationship in the large deformation was designed according to the published research [41], and sample dimension of shear test is shown in Figure 5a.

#### 2.2.4. FEM Simulation for the Shearing Process

1. Material property

The shearing process was simulated by the commercial software Abaqus. The elastic modulus and Poisson’s ratio are 210,000 MPa and 0.33 [42], respectively. The yield criterion of von Mises was applied in the simulation. Since the necking phenomenon occurs as the strength exceeds the tensile strength, the experimental stress–strain relationship in the large deformation is inaccurate. This problem is dealt with the method of shear test [43]. The same experimental method was applied in our study. The detailed procedure is summarized as follows: (i) The experimental data obtained in the uniaxial tensile test were fitted by the unsaturated model of Swift law and saturated model of Voce form, respectively. Additionally, the parameter of Swift law, including A, $\varepsilon_{0}$, n, shown in Equation (1), and the parameter of Voce form including $Q_{s}$, $Q_{0}$, $V_{0}$ expressed in Equation (2) are determined. (ii) The linear combined stress–strain relationship expressed in Equation (3) was input into the finite element model of shear test to determine the parameter h, and the parameter h was determined when the force–displacement curve in the simulation agrees well with the experimental curve, as shown in Figure 5b. The fitting results of hardening parameters are shown in Table 2.

(1) $$\sigma_{swift}({\bar{\varepsilon }}^{pl})=A{\left({\bar{\varepsilon }}^{pl}+\varepsilon_{0}\right)}^{n}$$

(2) $$\sigma_{voce}({\bar{\varepsilon }}^{pl})=Q_{s}-(Q_{s}-Q_{0})e^{\left[-\frac{V_{0}}{Q_{s}}{\bar{\varepsilon }}^{pl}\right]}$$

(3) $$\sigma ({\bar{\varepsilon }}^{Pl})=h\sigma_{swift}({\bar{\varepsilon }}^{Pl})+(1-h)\sigma_{voce}({\bar{\varepsilon }}^{pl})$$

2. Modeling and meshing

**Figure 6:**
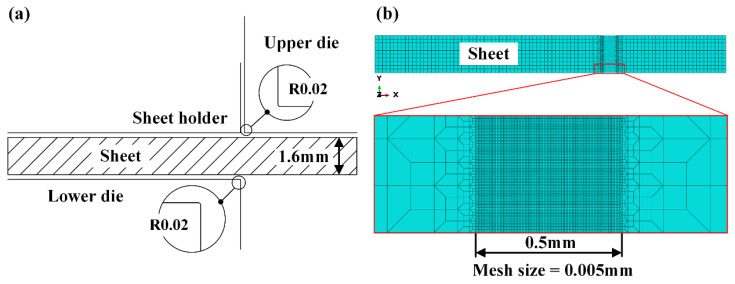
(**a**) Finite element model of shearing process. (**b**) Mesh strategy applied in the sheet.

The finite element model utilized in the simulation for the shearing process is shown in Figure 6a. The upper die, lower die and sheet holder were modeled as analytical rigid parts in the commercial software Abaqus. It should be noted that the differences of cutting edges of dies between the situations of experiment and simulation. For the experimental platform, the cutting edges of upper and lower dies were designed and fabricated without fillets, and the hardness of upper and lower dies was improved via appropriate heat treatment to keep the cutting edges sharply grounded in the whole experiment. For the simulation, the upper and lower dies were modeled with fillets to prevent the mesh penetration induced by the sharply angles of rigid parts. The specific value of the fillet was designed according to two aspects. On the one hand, the fillet values of upper and lower dies modeled as rigid parts should be large than the minimum mesh size in the sheet modeled as a deformable part. On the other hand, the magnitude of the upper and lower die fillet is in accordance with the existing literature [29]. Finally, the value of 0.02 mm was adopted in our study.

To reduce computation time and improve calculation accuracy at the same time, the local mesh refinement method is employed in the meshing for the sheet part, as shown in Figure 6b. The region far away from the shearing deformation zone was meshed into ten layers along the thickness direction with the size of 0.16 mm in each layer, which is accurate enough in the simulation for sheet forming without fracture. For predicting the morphology of the sheared edge, the range of mesh refinement was set to cover the width of the whole crack generated in the shearing process. The critical region’s fracture behavior was meshed at the size of 0.005 mm in the range of 0.5 mm, as shown in Figure 6b, which was determined after several simulation attempts.

3. Ductile fracture criteria

Six widely used uncoupled ductile fracture criteria are selected to simulate the shearing process of QP980 steel. The simulation results are compared with the corresponding experiment results to determine the optimum ductile fracture criterion. The ductile fracture criteria used in our study are listed in Table 3.

**Table 3:** Summary of ductile fracture criteria used in the research.

Ductile Fracture Criteria	Equations
Freudenthal [44]	$\int_{0}^{{\bar{\varepsilon }}_{D}}\bar{\sigma }d{\bar{\varepsilon }}^{pl}=c_{1}$
Cockcroft and Latham [45]	$\int_{0}^{{\bar{\varepsilon }}_{D}}\sigma_{1}d{\bar{\varepsilon }}^{pl}=c_{2}$
Rice and Tracey [46]	$\int_{0}^{{\bar{\varepsilon }}_{D}}0.283\exp\left(\frac{3\sigma_{m}}{2\bar{\sigma }}\right)d{\bar{\varepsilon }}^{pl}=c_{3}$
Brozzo [47]	$\int_{0}^{{\bar{\varepsilon }}_{D}}\frac{2\sigma_{1}}{3\left(\sigma_{1}-\sigma_{m}\right)}d{\bar{\varepsilon }}^{pl}=c_{4}$
Oh [48]	$\int_{0}^{{\bar{\varepsilon }}_{D}}\left(\frac{\sigma_{1}}{\bar{\sigma }}\right)d{\bar{\varepsilon }}^{pl}=c_{5}$
Oyane-Sato [49]	$\int_{0}^{{\bar{\varepsilon }}_{D}}\left(\frac{\sigma_{m}}{\bar{\sigma }}+c_{6}\right)d{\bar{\varepsilon }}^{pl}=c_{7}$

Note: ${\bar{\varepsilon }}^{pl}$ is the equivalent strain, ${\bar{\varepsilon }}_{D}$ is the equivalent plastic strain at the onset of damage, $\bar{\sigma }$ is the von Mises stress, $\sigma_{1}$ is the maximum principal stress, $\sigma_{m}$ is the hydrostatic stress, $c_{1}$, $c_{2}$, $c_{3}$, $c_{4}$, $c_{5}$, $c_{6}$, $c_{7}$ are the material constants, which are determined through inverse tensile curves fitting, and this method was introduced and verified in the study for the hot stamping steel [50]. The parameters of ductile criteria of QP980 steel are also obtained by the method in this paper, and the results are summarized in Table 4.

**Table 4:** Parameters in the six ductile criteria.

Material	$c_{1}$/MPa	$c_{2}$/MPa	$c_{3}$	$c_{4}$/MPa	$c_{5}$	$c_{6}$	$c_{7}$
QP980	230.53	213.49	0.089	0.19	0.20	0.17	2.08

**Figure 7:**
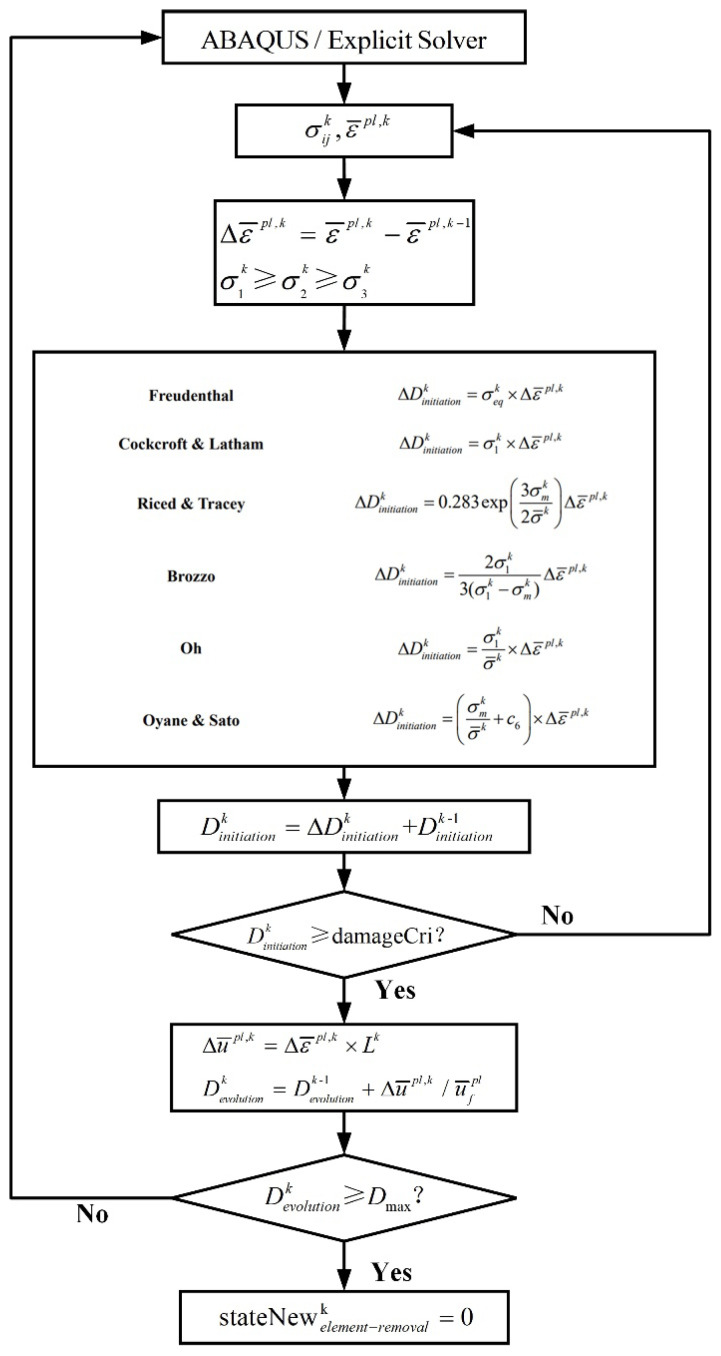
Flow chart of VUSDFLD subroutine implement.

The six ductile fracture criteria are newly implemented via developing the user-defined subroutine VUSDFLD in Abaqus for simulating the shearing process of QP980 steel, and the linear damage evolution method introduced in Abaqus is incorporated to compute the damage evolution after the crack initiation, as expressed in Equation (6). The process of implementing six uncoupled ductile fracture criteria is summarized by a flow chart, as shown in Figure 7.

(4) $${\dot{\bar{u}}}^{pl}=L\times {\dot{\bar{\varepsilon }}}^{pl}$$

(5) $${\dot{D}}_{evolution}=\frac{{\dot{\bar{u}}}^{pl}}{{\bar{u}}_{f}^{pl}}=\frac{L\times {\dot{\bar{\varepsilon }}}^{pl}}{{\bar{u}}_{f}^{pl}}$$

(6) $$D_{evolution}=\int_{0}^{{\bar{\varepsilon }}_{f}}\frac{L\times {\dot{\bar{\varepsilon }}}^{pl}}{{\bar{u}}_{f}^{pl}}d\bar{\varepsilon }$$

where ${\dot{\bar{\varepsilon }}}^{pl}$ is the plastic strain rate, L is the characteristic length, ${\dot{D}}_{evolution}$ is the damage evolution rate, ${\dot{\bar{u}}}^{pl}$ is the plastic displacement rate, ${\bar{\varepsilon }}_{f}$ is the equivalent plastic strain at fracture, and $G_{f}$ is fracture energy per unit area, which is given as

(7) $$G_{f}=\int_{{\bar{\varepsilon }}_{0}^{pl}}^{{\bar{\varepsilon }}_{f}^{pl}}L\sigma_{y0}d{\bar{\varepsilon }}^{pl}=\int_{0}^{{\bar{u}}_{f}^{pl}}\sigma_{y0}d{\bar{u}}^{pl}$$

Equation (7) [51] introduces the definition of the equivalent plastic displacement, ${\bar{u}}^{pl}$, $\sigma_{y0}$ is the value of the yield stress, where the equivalent plastic displacement at failure, and ${\bar{u}}_{f}^{pl}$, is computed as

(8) $${\bar{u}}_{f}^{pl}=\frac{2G_{f}}{\sigma_{y0}}$$

For QP980 steel used in this paper, $G_{f}=37.1\;\frac{N}{mm}$, $\sigma_{y0}=411.9\;MPa$.

The initial values of L, $D_{initiation}$ and $D_{evolution}$ are 0.005 mm, 0, 0, respectively. The fracture behavior is described in two parts. The first part is the onset of damage, which is described by the variable of $D_{initiation}$. The second part is the evolution of damage, which is described by the valuable of $D_{evolution}$. The calculation of damage evolution begins when the value of $D_{initiation}$ reaches damageCri. The solution-dependent state variable “stateNew” provided in Abaqus is used to determine whether the element meets the deletion condition.

## 3. Results and Discussion

### 3.1. Analysis of Deformation and Damage Evolution in the Shearing Process

#### 3.1.1. Shearing Deformation

**Figure 8:**
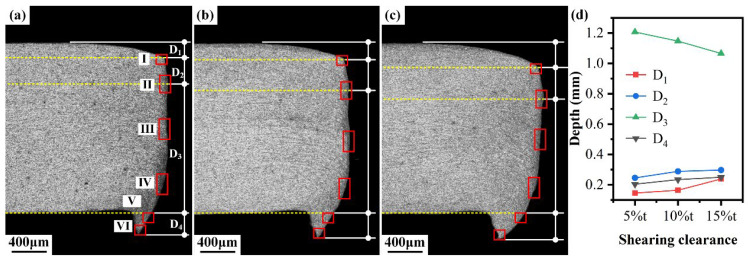
Morphology of sheared edge obtained under different shearing clearances and depths of typical zones: (**a**) 5%t; (**b**) 10%t; (**c**) 15%t. (**d**) Depths of rollover zone, burnish zone, fracture zone and burr zone.

The morphology of sheared edge in the cross section was observed along the thickness direction using an optical microscope (OM), as shown in Figure 8. Four typical zones, including rollover zone, burnish zone, fracture zone, and burr zone, were formed after the shearing process. In Figure 8, the boundaries of four typical zones are indicated by yellow dot lines, and the depths of these four zones were determined and are labeled as D1, D2, D3, and D4, respectively. To investigate the influence of shearing clearance on the morphology of the sheared edge, the four depths corresponding to the shearing clearances of 5%t, 10%t, and 15%t are compared in Figure 8d. It can be seen that the maximum depth always situates in the fracture zone among the four typical zones. In addition, there are more microdefects distributed in the fracture zone than others since the fracture zone is formed by the propagation of shearing cracks. Therefore, it can be inferred that the fracture zone has the greatest impact on the formability of the sheared edge, this result will be verified in the next analysis. For the effect of shearing clearance on other typical zones, it can be observed that the depth of the rollover zone, burnish zone and burr zone increases with the increase in the shearing clearance except the fracture zone. In the case of large shearing clearance, more deformation occurs in the rollover zone and burnish zone, which increases their depth. Meanwhile, increasing the shearing clearance delays the initiation of the shearing crack, which reduces the depth of the fracture zone [52].

**Figure 9:**
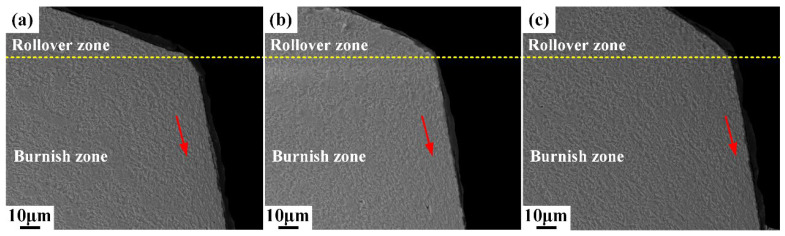
Microstructure of sheared edge in the area I obtained under different shearing clearances: (**a**) 5%t; (**b**) 10%t; (**c**) 15%t.

To further investigate the damage induced during the shearing process, six microscopic areas in the sheared edge labeled as I, II, III, IV, V, and VI with red rectangles in Figure 8 are enlarged for the comprehensive observation using scanning electron microscope (SEM) technology. Obvious plastic flow occurs in area I, the direction is along the movement of upper die, as shown in Figure 9. Additionally, no microdefects that appeared in area I, which means that the deformation degree in the rollover zone and upper burnish zone is lower than the forming limit.

**Figure 10:**
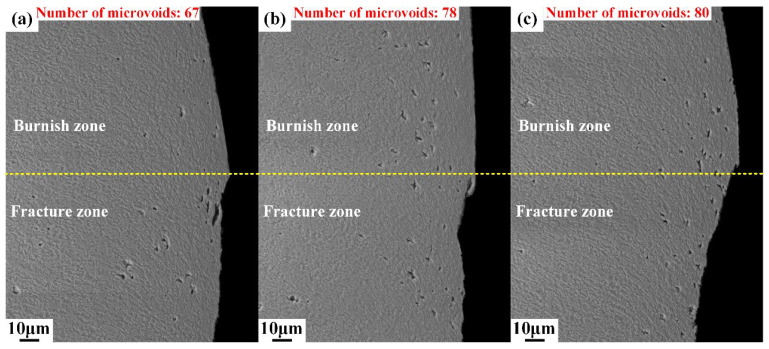
Microstructure of sheared edge in area II, obtained under different shearing clearances: (**a**) 5%t; (**b**) 10%t; (**c**) 15%t.

**Figure 11:**
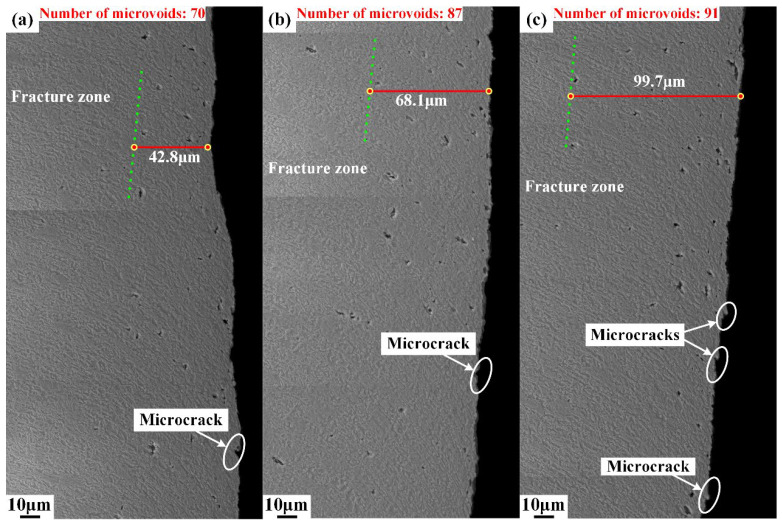
Microstructure of sheared edge in area III obtained under different shearing clearances: (**a**) 5%t; (**b**) 10%t; (**c**) 15%t.

**Figure 12:**
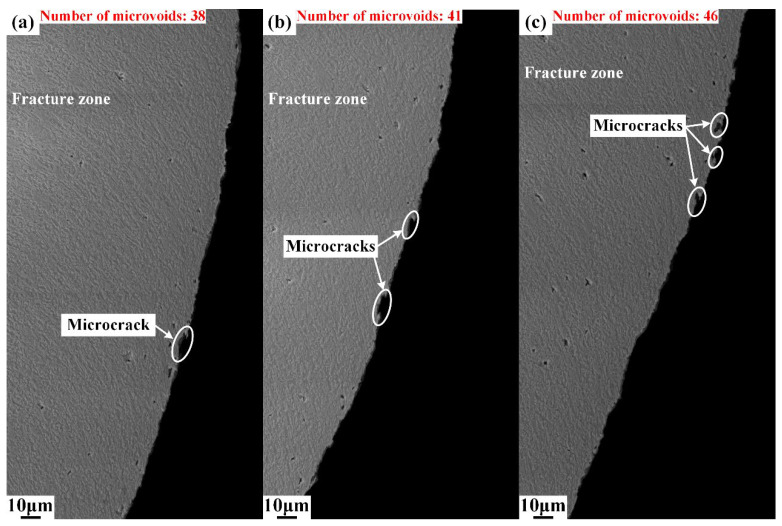
Microstructure of sheared edge in area IV obtained under different shearing clearances: (**a**) 5%t; (**b**) 10%t; (**c**) 15%t.

**Figure 13:**
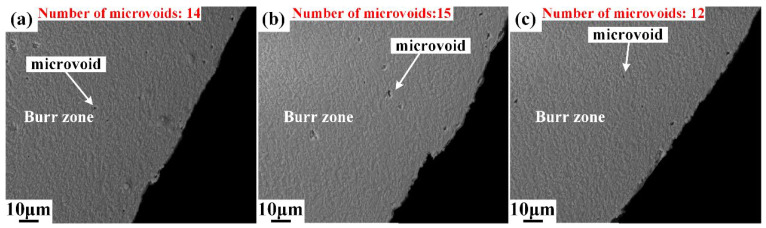
Microstructure of sheared edge in area V obtained under different shearing clearances: (**a**) 5%t; (**b**) 10%t; (**c**) 15%t.

**Figure 14:**
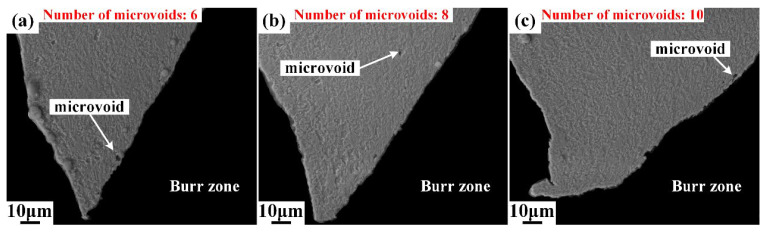
Microstructure of sheared edge in Area VI obtained under different shearing clearances: (**a**) 5%t; (**b**) 10%t; (**c**) 15%t.

Different from area I, plenty of microvoids emerge in area II, as shown in Figure 10. The deformation degree intensified as the upper die continued to move downward, and the cutting edge of the upper die began to penetrate into the steel sheet after the rollover zone was formed. When the depth of penetration researches a certain level, the microvoids start to generate in the conjunction of the lower part of the burnish zone and upper part of the fracture zone, as shown in Figure 10, and the boundary of the burnish zone and fracture zone is indicated by the yellow dot line. The number of microvoids in area II was counted, and the results show that the number of microvoids increased from 67 to 80, with the shearing clearance increasing from 5%t to 15%t. This indicates that the damage of microvoids firstly initiates in the lower part of burnish zone and grows with the increase in shearing clearance. Additionally, the sheared surfaces of the burnish zone and fracture zone are totally different. The sheared surface in the burnish zone is smooth, while the sheared surface is rough in the fracture zone due to the distribution of V-shape notches, which are called microcracks in this paper. The typical microcracks are labeled in Figure 11 and Figure 12. These microcracks can become crack sources in the subsequent deformation, which can directly lead to crack propagation. Therefore, compared with other typical zones, the fracture zone is prone to cracking first. The microvoids are also generated in the burr zone, as shown in Figure 13 and Figure 14. Different from microvoids in the fracture zone, the number of microvoids in the burr zone reduces rapidly. On the one hand, the area of the burr zone is small. On the other hand, the burr zone is the final deformation zone in the process of shearing crack propagation. Crack propagation resistance decreases with crack propagating due to the reduction in the thickness of the uncracked sheet. Therefore, the degree of deformation in the burr zone decreases owing to the reduction in crack propagation resistance, and a few microvoids are generated in the burr zone. Additionally, the number of microvoids generated in areas II, III, IV, V, and VI is displayed in Figure 10, Figure 11, Figure 12, Figure 13 and Figure 14. It can be seen that the number of microvoids in all areas increases with the increasing shearing clearance except for area V. This difference highlights that the results obtained by analyzing local areas cannot clearly point out the relationship between the generation of microvoids and shearing clearance. Therefore, the microvoids in the whole sheared edge are fully counted and analyzed in the next section.

#### 3.1.2. Analysis of Microvoids Generated in the Shearing Process

**Figure 15:**
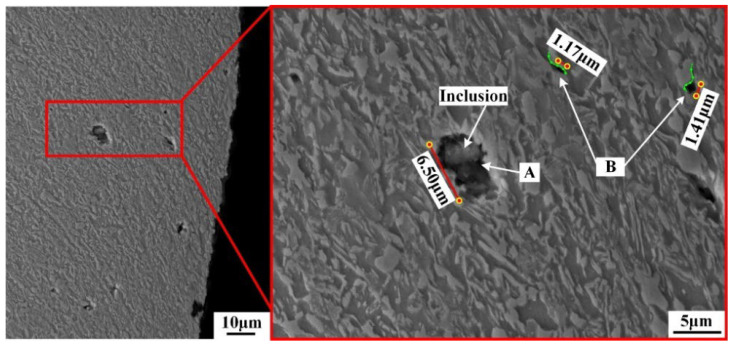
Location of microvoids in the sheared edge. (A represents the microvoid generated from the inclusion; B represents the microvoids formed at the phase interfaces; the green lines represent the phase interfaces.)

The nucleation site, number, and size of microvoids obtained under different shearing clearances were carefully compared and analyzed to systematically investigate the damage of microvoids. Two different types of microvoids were observed in the sheared edge, as shown in Figure 15. One was formed at the phase interfaces, and the other was generated from the inclusion. The elements and contents of inclusion are shown in Table 5, determined by a semi-quantitative analysis using EDS (Energy Dispersive Spectroscopy). The size of the microvoid generated from the inclusion is 6.50 μm, which is obviously larger than the sizes of 1.37 μm and 1.41 μm, corresponding to the microvoids formed at the phase interfaces. The emergence of large-sized microvoids generated from the inclusion suggests that the influence of the cleanliness of steel on the damage initiation in the shearing process should be carefully considered.

**Figure 16:**
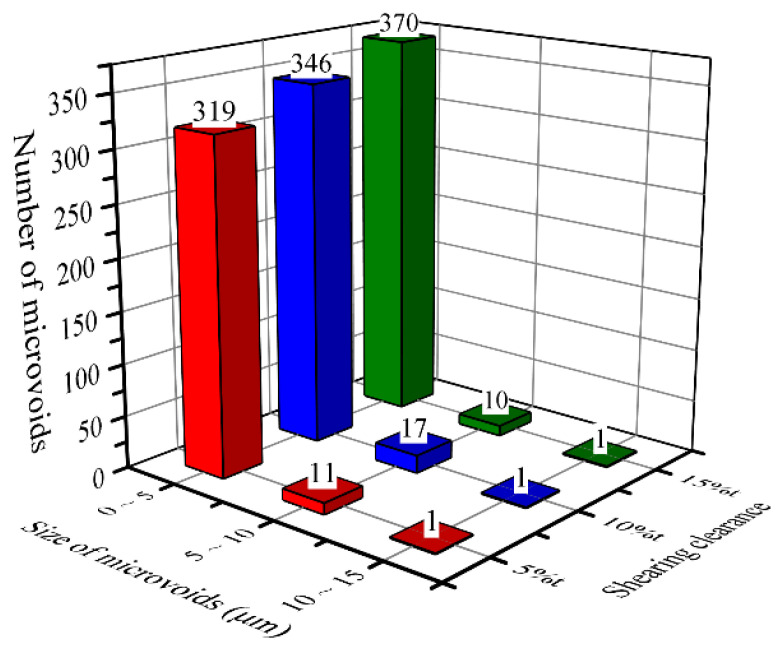
Number of microvoids generated in the sheared edge under the shearing clearances of 5%t, 10%t, and 15%t.

The number of microvoids was counted in the whole sheared edge. Three ranges, including 0–5 μm, 5–10 μm, and 10–15 μm, were selected to record the number of microvoids, as shown in Figure 16. It can be observed that the majority of microvoids range in size from 0 to 5 μm. Additionally, the number of microvoids with a size from 0 to 5 μm increases with the shearing clearance. However, the situation of the microvoids with sizes of 5 μm to 10 μm and 10 μm to 15 μm is different. This is mainly because the large microvoids with a size of more than 5 μm are primarily generated from the inclusions, which are always randomly distributed and small in number; thereby, the number of microvoids with a large size more than 5 μm does not have a clear relationship with the shearing clearance.

**Figure 17:**
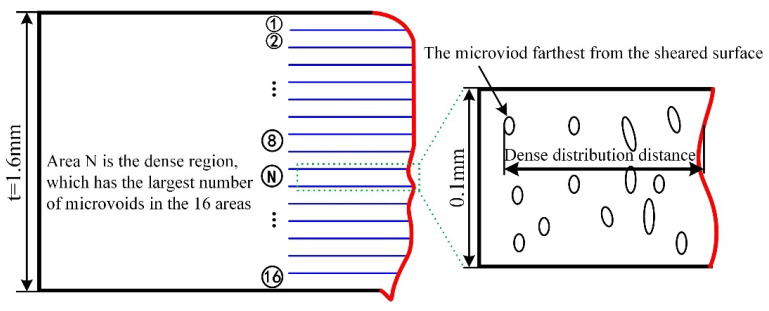
Method of determining the dense distribution distance.

**Figure 18:**
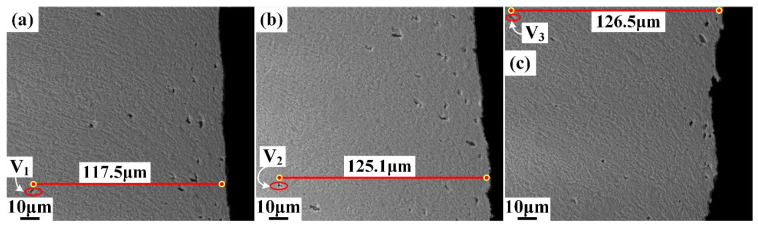
Farthest microvoid distributed in the sheared edge: (**a**) 5%t; (**b**) 10%t; (**c**) 15%t.

Two kinds of distance were selected to characterize the distribution of microvoids generated under the three shearing clearances. One is the dense distribution distance. The method of determining the distance is shown in Figure 17. The cross section of the sheared edge was divided into 16 equal areas along the thickness direction to determine the dense region, which is the area with the largest number of microvoids in the 16 areas. Then, the dense distribution distance was measured from the farthest microvoid to the sheared surface in the dense region. The measured result of this distance is shown in Figure 11, and the other is the maximum distance from the farthest microvoid to the sheared surface along the horizontal direction, as shown in Figure 18.

The dense region of microvoids is indicated by the green dot line, as shown in Figure 11, and the dense distribution distance of microvoids was measured and labeled. It can be seen that the dense region of microvoids is located in the fracture zone and the dense distribution distance increases with the increasing shearing clearance. In Figure 18, the farthest microvoids generated under the three shearing clearances are labeled as V1, V2, and V3, respectively. With the increase in the shearing clearance, the range of the maximum distance of microvoids increased from 117.5 μm to 126.5 μm.

#### 3.1.3. Study of Microcracks Formed in the Shearing Process

**Figure 19:**
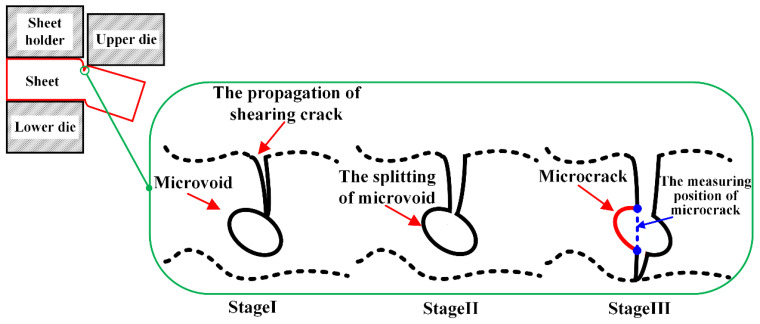
Process of microcrack generation.

Plenty of V-shape micronotches are distributed in the sheared surface of fracture zone, as shown in Figure 11 and Figure 12. As mentioned before, these micronotches are called microcracks in this paper. The formation mechanism of microcracks is illustrated in Figure 19. In Section 3.1.1, we point out that microvoids are firstly initiated at the boundary of to burnish zone and fracture zone. As the deformation continues, the growing microvoids gradually coalesce to the shearing crack, which is indicated in stage I in Figure 19, and then the microvoids are split by the propagation of the shearing crack, as shown in stage II in Figure 19. Finally, the V-shape micronotches form and distribute in the sheared surface. In addition, the measuring position to determine the size of the microcrack is shown in stage III in Figure 19 with the blue dot line.

**Figure 20:**
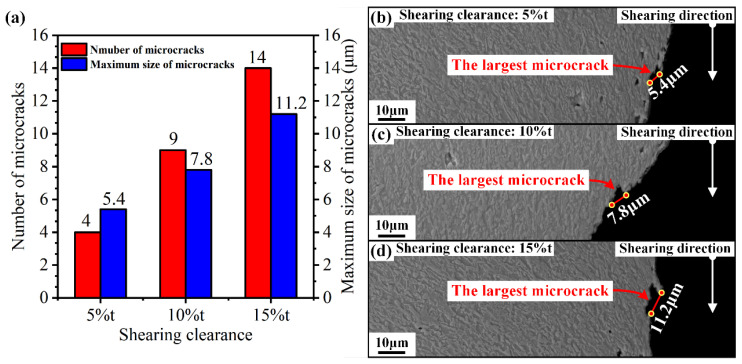
(**a**) Number and size of microvoids generated in the sheared edge. (**b**) largest microcrack obtained under the shearing clearances of 5%t, (**c**) 10%t, (**d**) 15%t.

Furthermore, the number and maximum size of microcracks in the whole sheared edge obtained under the shearing clearances of 5%t, 10%t, and 15%t are shown in Figure 20a. It can be observed that the number of microcracks increased with the increasing shearing clearance. For the maximum size of microcracks, it increased from 5.4 μm to 7.8 μm to 11.2 μm, as the shearing clearance increased from 5%t to 10%t, to 15%t, and the corresponding maximum size microcracks are shown in Figure 20b–d. Compared with the damage of microvoids, the size of microcracks is greater than that of microvoids in all shearing conditions investigated in our study. More importantly, the microcracks are distributed on the sheared surface. When these microcracks are loaded, they can cause crack propagation at any time. This indicates that microcracks have a greater impact on the formability of sheared edges than microvoids. Controlling the damage degree of microcracks is crucial for reducing the risk of sheared-edge cracking.

#### 3.1.4. Characterization of Work-Hardening Behavior

After investigating the microdefects generated in the sheared edge, the sheared affected zone was characterized according to the working hardening behavior, which was measured by the microhardness test. Before the test was performed on the sheared edge, the microhardness in the undeformed matrix was firstly obtained after eight microhardness experiments. The lowest and highest microhardnesses are 310 HV and 325 HV, respectively, and the average microhardness is around 317.5 HV. The sheared affected zone was determined as the microhardness in the sheared edge decreased to the matrix hardness.

**Figure 21:**
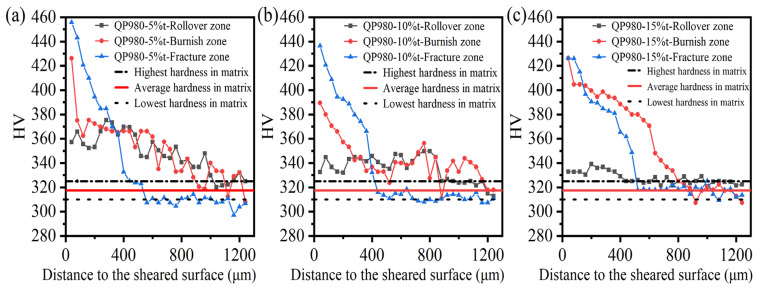
Microhardness distribution of sheared edge under different shearing clearances. (**a**) 5%t, (**b**) 10%t, (**c**) 15%t.

**Figure 22:**
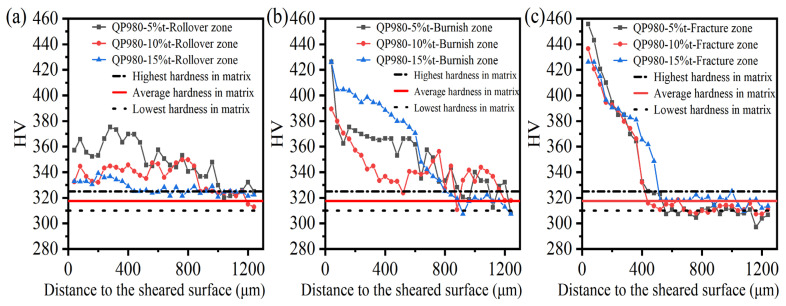
Microhardness distribution of sheared edge in different zones (**a**) rollover zone, (**b**) burnish zone, (**c**) fracture zone.

The testing position of microhardness in the sheared edge is illustrated in Figure 4, and the measured results are shown in Figure 21 and Figure 22. It can be perceived that distinct work-hardening behavior happens in the sheared edge. Additionally, the transformation of retained austenite into martensite during the shearing process promotes the increase in microhardness [53,54]. In general, the microhardness decreases with the increase in distance to the sheared surface. For the tested results of microhardness in different typical zones under the same shearing clearance, the weakest work hardening was located in the rollover zone among all shearing conditions performed in our study. This result is consistent with the discussion in Section 3.1.1 about the microstructure characterization, which indicates that the deformation degree in the rollover zone is minimum; thereby, the degree of damage in the rollover is also minimum. The highest microhardness always occurs in the fracture zone under the same shearing clearance. This is mainly associated with the deformation procedure. Generally, the initiation of shearing crack implies that the material has experienced the maximum plastic deformation, which means that the degree of work-hardening behavior reaches the highest level in the fracture zone.

Figure 22 shows the microhardness in the rollover zone, burnish zone, and fracture zone with different shearing clearances, and it can be seen that the decreasing trends of microhardness in the three typical zones are totally different. The hardening trend in the rollover zone is the smoothest among all typical zones in the sheared edge. The hardened area in the burnish zone has the largest distance, 800 μm, while the maximum microhardness occurs in the fracture zone. It can be inferred that the plastic deformation mainly occurs in the burnish zone before the generation of shearing crack. As the shearing crack propagates in the fracture zone, the microstructure is rapidly hardened with a short influence range. Combined with the former analysis about the damage of microcracks distributed in the sheared surface of the fracture zone, it can be found that the propagation of shearing cracks not only results in the generation of microcracks but also leads to severe work-hardening behavior in the fracture zone simultaneously.

In this section, three kinds of damage, including microvoids, microcracks, and work-hardening behavior, are determined and investigated. From the observation of the dense distribution area of microvoids, the microcracks serving as crack sources, and the highest microhardness, all occur in the fracture zone, it can be seen that the damage degree in the fracture zone is the most serious. Therefore, the key point in improving the formability of sheared edges is mainly to reduce the depth of the fracture zone and control the damage induced during the shearing process.

### 3.2. Finite Element Analysis of Shearing Process

**Figure 23:**
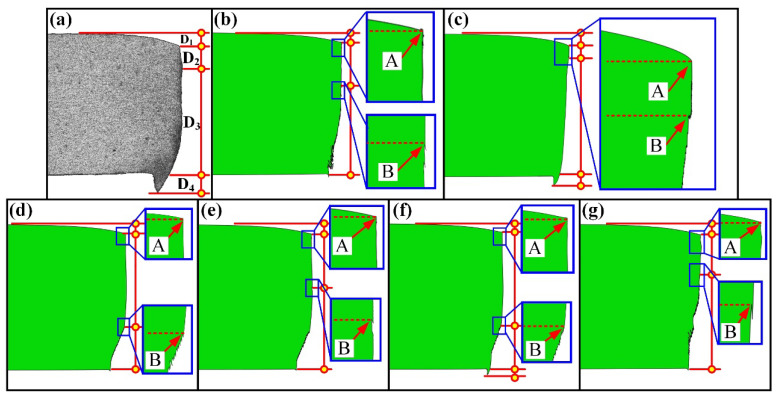
The morphology of sheared edge obtained under the shearing clearance of 5%t. (**a**) Experimental results. (**b**) Simulation results of Freudenthal model. (**c**) Cockcroft and Latham model. (**d**) Rice and Tracey model. (**e**) Brozzeo model. (**f**) Oh model. (**g**) Oyane-Sato model.

**Figure 24:**
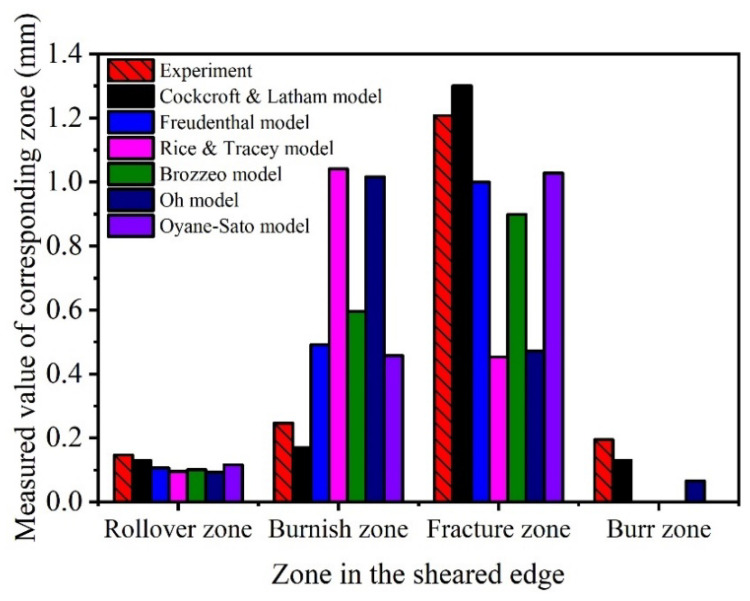
Depth of rollover zone, burnish zone, fracture zone, and burr zone measured from the experiment results and simulation results simulated with different ductile fracture criteria.

The damage generated in the sheared edge was analyzed in Section 3.1 based on experiments. In addition, it is necessary to analyze the distribution of strain and stress in the shearing process. Due to the variation of stress and strain in the whole shearing deformation being difficult to determine through experimental methods, the finite element analysis was introduced in this study. Considering that the shearing deformation involves the generation of shearing crack, the ductile fracture criterion needs to be considered in the constitutive model to describe the fracture behavior of QP980 steel. In order to identify the optimum ductile fracture criterion for the simulation of shearing process of QP980 steel, six ductile fracture criteria including Freudenthal, Cockcroft and Latham, Rice and Tracey, Brozzeo, Oh, and Oyane-Sato model are implemented in Abaqus by developing the user-defined subroutine VUSDFLD. The corresponding results simulated under the shearing clearance of 5% t are compared in Figure 23. It can be seen that only the results of the Cockcroft and Latham and Oyane-Sato models have successfully simulated the burr zone. The depth of the rollover zone, burnish zone, fracture zone, and burr zone previously used in Section 3.1.1 were adopted again for evaluating the simulation results of different ductile fracture criteria. The four typical zones in this simulation were determined according to the morphology. Take Figure 23c as an example to illustrate the specific determination method. The bending part in the upper surface of the sheet is the rollover zone, which has an obvious ending point, as indicated by A in Figure 23c. In other words, point A is the dividing point between the rollover zone and burnish zone. The edge of the burnish zone is smooth, which was verified in our experiment. Therefore, the first serration indicated by B in Figure 23c is the dividing point between the burnish zone and fracture zone. The lower surface of sheet is the boundary between fracture zone and burr zone, so the burr zone is the part exceeding the lower surface of the original sheet. The determined four depths are labeled as D1, D2, D3, and D4, respectively, as shown in Figure 23. The detailed measured values of corresponding zones are compared in Figure 24. It can be shown that the simulation results of the Cockcroft and Latham model match best with the experiment results in all typical zones compared with other damage models. Therefore, the Cockcroft and Latham model was selected for the next simulation analysis.

**Figure 25:**
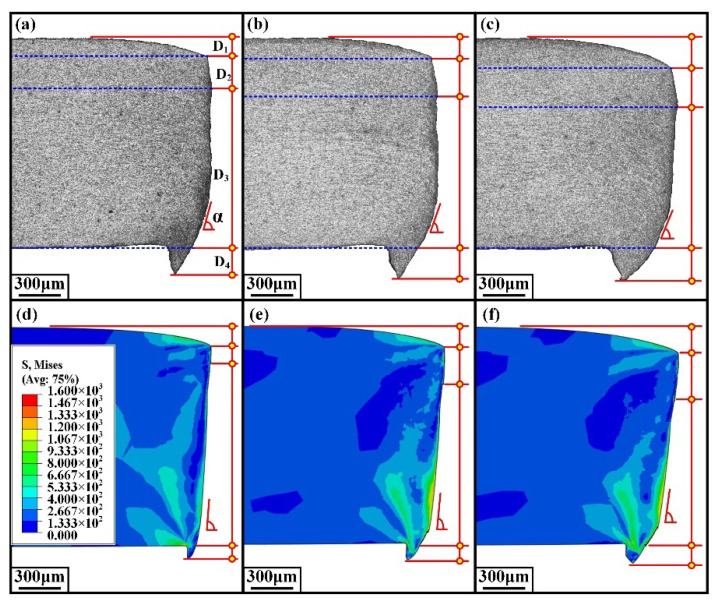
Experiment and simulation results of sheared edge. (**a**–**c**) Experiment results sheared at 5%t, 10%t, and 15%t. (**d**–**f**) Simulation results sheared at 5%t, 10%t, and 15%t.

The morphology of sheared edge simulated through the Cockcroft and Latham model under the shearing clearances of 5%t, 10%t, and 15%t is shown in Figure 25d–f, respectively. Additionally, the corresponding experiment results are shown in Figure 25a–c. The indexes of D1 to D4 utilized to evaluate the accuracy of six damage models were also adopted to estimate the simulation result of the Cockcroft and Latham model. Additionally, the index α represents the angle between the edge of the fracture zone and the horizontal direction was added to more comprehensively assess the simulation error of the Cockcroft and Latham model under different shearing clearances.

**Figure 26:**
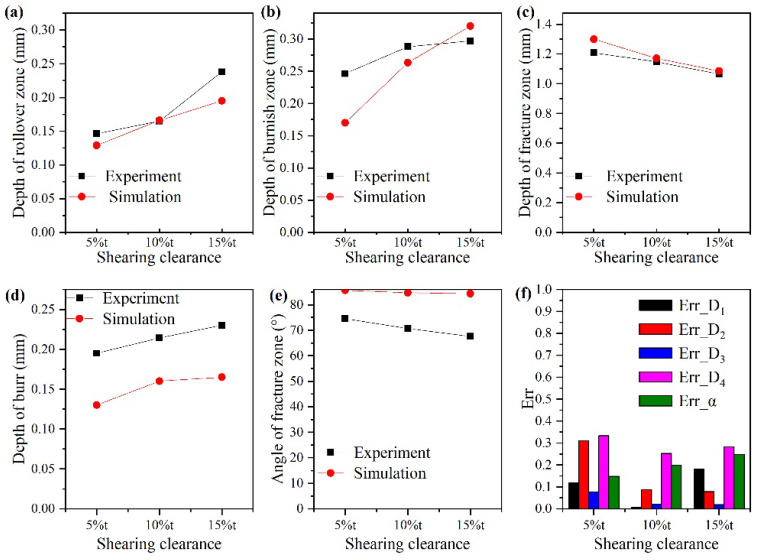
Analysis of simulation error. (**a**) Experiment and simulation results of rollover zone depth. (**b**) Experiment and simulation results of burnish zone depth. (**c**) Experiment and simulation results of fracture zone depth. (**d**) Experiment and simulation results of burr depth. (**e**) Experiment and simulation results of fracture zone angle. (**f**) Error calculation.

The experimental and simulated values of these five indexes are shown in Figure 26a–e. It can be recognized that the variation trend of simulation results matches well with that of the experimental results among all five evaluating indexes. The error between the simulation and experiment was calculated according to Equation (9), and the result is shown in Figure 26f. It can be observed that all errors are below 25% expect for the error of the depth of the burr zone. This is mainly because the generation process of the burr zone is a complex deformation process. Although the discrete fine meshes were adopted to describe the ductile fracture behavior of the continuum material, the mesh itself has an influence on the element deletion technology, which is used to simulate the material separation in the finite element analysis.

(9) $$err_{i}=\left|\frac{i^{\mathrm{experiment}}-i^{\mathrm{simulation}}}{i^{\mathrm{experiment}}}\right|,i=D_{1},D_{2},D_{3},D_{4},\alpha$$

where $i^{\mathrm{experiment}}$ is the experiment value and $i^{\mathrm{simulation}}$ is the simulation value.

**Figure 27:**
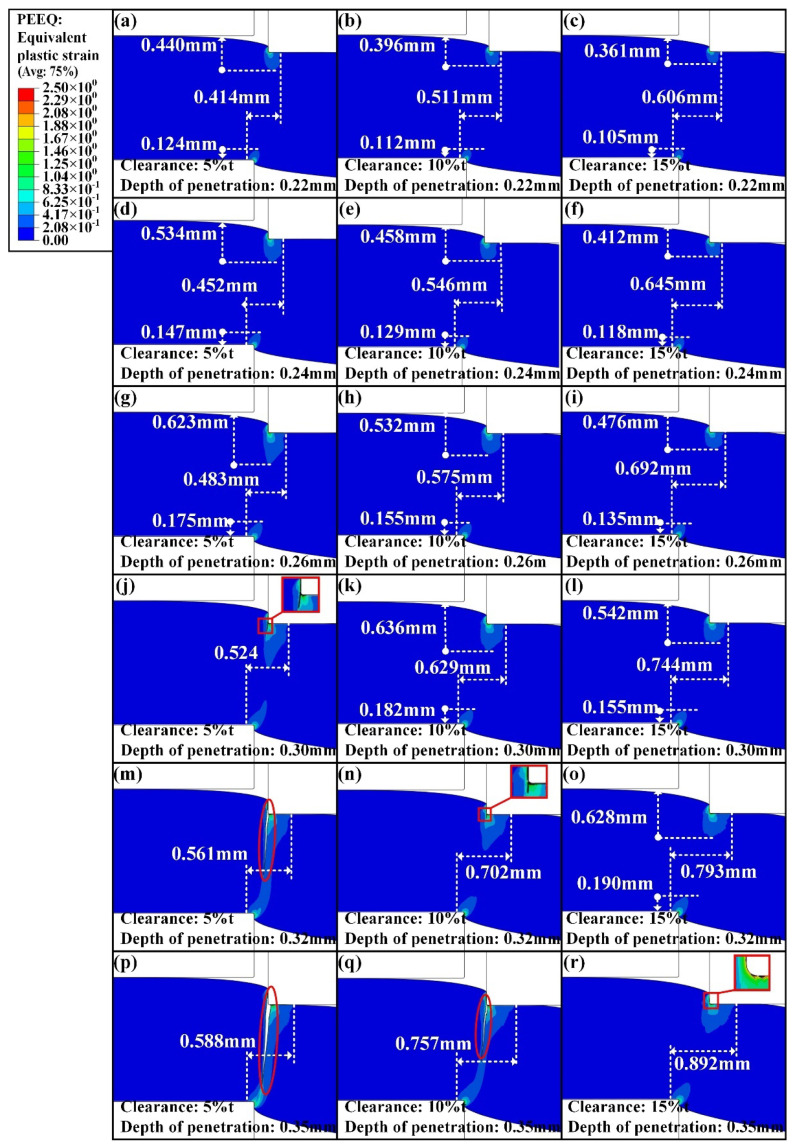
Strain distribution under the shearing clearances of 5%t, 10%t, and 15%t at different stages during the shearing process. (**a**–**c**) Depth of penetration = 0.13 mm. (**d**–**f**) Depth of penetration = 0.15 mm. (**g**–**i**) Depth of penetration = 0.17 mm. (**j**–**l**) Depth of penetration = 0.24 mm. (**m**–**o**) Depth of penetration = 0.26 mm. (**p**–**r**) Depth of penetration = 0.29 mm.

**Figure 28:**
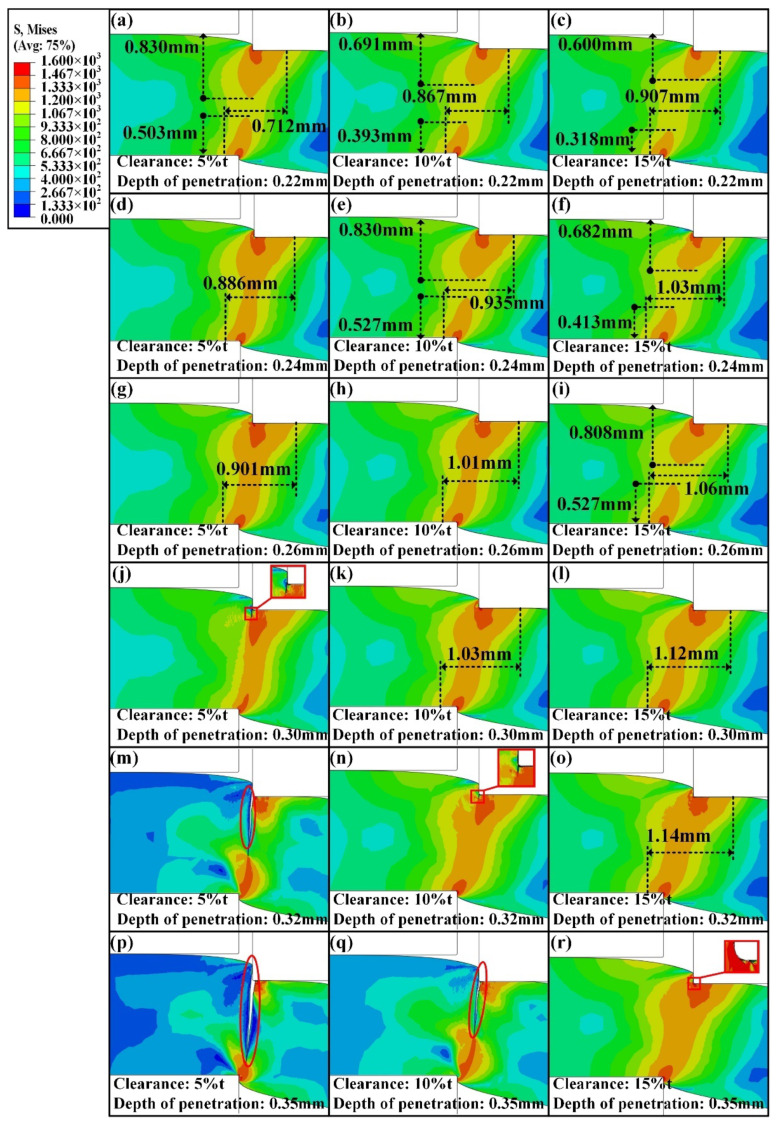
Stress distribution under the shearing clearances of 5%t, 10%t, and 15%t at different stages during the shearing process. (**a**–**c**) Depth of penetration = 0.13 mm. (**d**–**f**) Depth of penetration = 0.15 mm. (**g**–**i**) Depth of penetration = 0.17 mm. (**j**–**l**) Depth of penetration = 0.24 mm. (**m**–**o**) Depth of penetration = 0.26 mm. (**p**–**r**) Depth of penetration = 0.29 mm.

For further investigation, the whole shearing deformation was analyzed according to the simulation results. The variation of the strain and stress distribution corresponding to the penetration depths of 0.13 mm, 0.15 mm, 0.17 mm, 0.24 mm, 0.26 mm, and 0.29 mm were compared, as shown in Figure 27 and Figure 28. At the beginning of shearing deformation, the deformation primarily occurs in the contact area between the cutting edges of dies and the sheet. As the upper die continues to move, the deformation gradually propagates the whole thickness.

To quantitatively analyze the variation of strain distribution, the equivalent plastic strains in the shearing deformation under the three shearing clearances corresponding to the six depths of penetration are compared in Figure 27. Three distances are used to measure the equivalent plastic strain distribution, the distance of strain caused by contact with the upper die to the upper surface of the sheet along the thickness direction, the distance of strain derived from contact with the lower die to the lower surface of sheet along the thickness direction, and the distance of two strain areas along the horizontal direction. The value of 2.08 × 10^−1^ for the equivalent plastic strain was used to define the three kinds of distances. The measured results are labeled in Figure 27a–r, and it can be seen that the range of strain distribution along the horizontal direction increases with increasing shearing clearance under the same depth of penetration. The increase in the range of strain distribution means that the influenced area is increased, which results in the increase in damage induced by the shearing process. In addition, it can be observed that the speed of strain propagating through the whole thickness increases as the shearing clearance decreases. Therefore, the shearing crack is generated earlier under the shearing clearance of 5%t than that under the shearing clearances of 10%t and 15%t, as shown in Figure 27j,n,r.

The stress distribution was also analyzed and measured through a similar method, as shown in Figure 28. Compared with the distribution of the stress at the six penetration depths, the value of stress of 1333 MPa is valid for a comparison to the change in stress distribution. In the horizontal direction, the area of stress distribution increases with the increasing shearing clearance. In the thickness direction, the stress spreads quickly as the shearing clearance decreases. In addition, it can be seen that decreasing shearing clearance contributes to obtaining a flat sheared edge and improving the formability of the sheared edge.

### 3.3. Evaluation of Tensile Property of Sheared Edge

**Figure 29:**
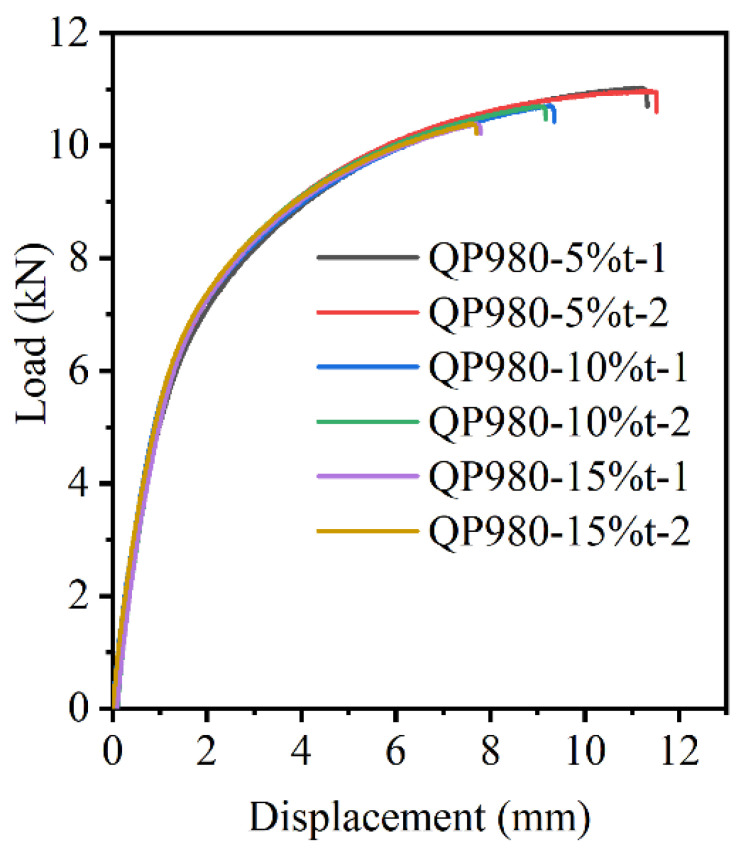
Load–displacement curves.

Finally, the tensile properties of the sheared edge obtained under the three shearing clearances were estimated by a tensile test, as shown in Figure 29. It can be seen that the results of tensile tests are in line with the former analysis about the microstructure evolution in the sheared edge. The formability of the sheared edge decreases with the increase in shearing clearance. This is mainly because increasing the shearing clearance aggravates the damage degree in the sheared edge. Therefore, controlling the shearing clearance is an effective method to improve the formability of the sheared edge by suppressing the damage degree.

## 4. Conclusions

In this paper, the mechanical damage induced during the shearing process of QP980 steel is experimentally investigated by a microstructure characterization and micro-/macromechanical property evaluation. In addition, the optimum uncoupled ductile fracture criterion was determined and employed to simulate the shearing deformation of QP980 steel. The conclusions are presented in the following:

(1) Three kinds of damage generated by mechanical deformation in the shearing process including microvoids, microcracks, and work-hardening behavior were determined, and the damage degree increased with the increasing shearing clearance. The optimum formability of the sheared edge with minimal damage validated by the tensile test was obtained under the shearing clearance of 5%t.
(2) Two different types of microvoids were identified based on the nucleation site. A large number of microvoids with small sizes (≤5 μm) were formed at phase interfaces, and a few microvoids generated from inclusions had large sizes of more than 5 μm. The V-shape microcrack evolved from the microvoid distributed in the sheared surface, which is a crucial factor for it can result in edge failure in the next forming process.
(3) Obvious work-hardening behavior appeared in the sheared edge. The longest work-hardening distance (800 μm) was located in the burnish zone, while the highest microhardness (>420 HV) occurred in the fracture zone, which further increases the risk of sheared-edge cracking. Therefore, reducing the depth of the fracture zone and controlling the damage degree are the key to improving the formability of sheared edges.
(4) Six widely applicated uncoupled ductile fracture models, including Freudenthal, Cockcroft and Latham, Rice and Tracey, Brozzeo, Oh, and Oyane-Sato models, were successfully introduced to simulate the shearing process through developing user-defined subroutine VUSDFLD in Abaqus. The simulation results of the Cockcroft and Latham model agree with experimental results better than others. The determination of suitable ductile fracture criteria for QP980 steel and accurate simulations are useful for engineers to shorten the time and cost of designing an optimum shearing process.

## Figures and Tables

**Table 1:** Mechanical properties of QP980 steel.

Material	Thickness/mm	Yield Strength/MPa	Tensile Strength/MPa	Total Elongation/%
QP980	1.6	606.4	1098.7	21.4

**Table 2:** Hardening parameters.

Material	A/MPa	$\varepsilon_{0}$	n	$Q_{s}$/MPa	$Q_{0}$/MPa	$V_{0}$	h
QP980	1967.75	0.0025	0.22	1296.54	576.63	25,889.67	0.70

**Table 5:** Elements and contents of inclusion.

Element	Weight (%)	Atomic (%)
O	60.15	74.19
Al	14.02	10.26
S	23.02	14.17
Ca	2.81	1.38

## Data Availability

All data included in this study are available upon request by contact with the corresponding author.
